# USP2a alters chemotherapeutic response by modulating redox

**DOI:** 10.1038/cddis.2013.289

**Published:** 2013-09-26

**Authors:** B Benassi, M Marani, M Loda, G Blandino

**Affiliations:** 1 Unit of Radiation Biology and Human Health, ENEA-Casaccia, Rome, Italy; 2 Molecular Medicine Department, Italian National Cancer Institute Regina Elena, Rome, Italy; 3 Department of Medical Oncology and Center for Molecular Oncologic Pathology, Dana-Farber Cancer Institute, Harvard Medical School, Boston, MA, USA; 4 Division of Cancer Studies, King's College London, London, UK; 5 Translational Oncogenomics Unit, Italian National Cancer Institute Regina Elena, Rome, Italy

**Keywords:** USP2a, miR-34b/c, c-Myc, GSH, chemotherapy, prostate cancer

## Abstract

Cancer cells are characterized by altered ubiquitination of many proteins. The ubiquitin-specific protease 2a (USP2a) is a deubiquitinating enzyme overexpressed in prostate adenocarcinomas, where it exhibits oncogenic behavior in a variety of ways including targeting c-Myc via the miR-34b/c cluster. Here we demonstrate that USP2a induces drug resistance in both immortalized and transformed prostate cells. Specifically, it confers resistance to typically pro-oxidant agents, such as cisplatin (CDDP) and doxorubicin (Doxo), and to taxanes. USP2a overexpression protects from drug-induced oxidative stress by reducing reactive oxygen species (ROS) production and stabilizing the mitochondrial membrane potential (ΔΨ), thus impairing downstream p38 activation and triggering of apoptosis. The molecular mediator of the USP2a protective function is the glutathione (GSH). Through miR-34b/c-driven c-Myc regulation, USP2a increases intracellular GSH content, thus interfering with the oxidative cascade triggered by chemotherapeutic agents. In light of these findings, targeting Myc and/or miR-34b/c might revert chemo-resistance.

Deubiquitinating enzymes (DUBs) represent one of the largest families of proteins responsible for regulating the ubiquitin–proteasome system by opposing ubiquitination.^1^ Although DUBs have been first identified for their role in protein stabilization, in recent years several other mechanisms have emerged by which the balance ubiquitination/deubiquitination can regulate protein level, activity and subcellular location, thus driving different cellular processes from gene transcription and DNA repair to cell cycle and apoptosis.^2^

Alteration in the ubiquitination pattern is observed in most cancer cells, where it is manifested by destabilization of tumor suppressors and activation of oncogenes. This justifies the efforts for developing proteasome inhibitors in cancer therapy, like Bortezomib used successfully in myeloma, neuroblastoma and mesothelioma therapy.^3, 4^ DUB enzymes and proteasome activity also affect cancer chemo- and radio-resistance. In fact, proteasome inhibitors have been proposed to be used in combination with conventional antineoplastic agents. Therapeutic strategies are currently being investigated to overcome chemo-resistance based on the mechanisms of ubiquitination/deubiquitination.^5, 6, 7, 8, 9^ However, despite both the *in vitro* and *in vivo* (clinical) evidence of beneficial antineoplastic effects, the reason why blocking proteasomal degradation in a ‘non-specific' manner results in differential killing of tumor cell remains elusive.

In prostate, USP2a is a deubiquitinating enzyme specifically overexpressed in about 40% of prostate adenocarcinomas.^10, 11^ Its oncogenic behavior is ascribed to its interaction with and prevention of proteosomal degradation of specific protein targets involved in different cellular pathways, such as fatty acid synthase (FASN), Mdm2, MdmX, AIF, Cyclin D, Aurora-A and EGFR.^12, 13, 14, 15, 16, 17, 18^ Moreover, we recently reported that USP2a is a master regulator of microRNA (miRNA or miR) expression in both immortalized and transformed prostate epithelial cells, where its overexpression specifically regulates the miR-34b/c cluster to target c-Myc.^19^

miRNAs have been shown to be differently expressed in benign compared with malignant prostate tissue and in different stages of prostatic carcinogenesis.^20, 21, 22^ In addition, certain miRs have been found to predict and affect sensitivity to anticancer treatment.^23^ For instance, the tumor suppressor miR-148a attenuates paclitaxel resistance in PC3 cells,^24^ while overexpression of miR-143 suppresses prostate proliferation and migration, and increases sensitivity to docetaxel (DTX) by targeting the EGFR/RAS/MAPK pathway.^25^ miR-34 family members in particular have been often associated to cancer resistance. In prostate cancer, ectopic miR-34 expression triggers cell cycle arrest and growth inhibition and attenuates chemo-resistance to camptothecin (CPT), Doxo and paclitaxel exposure.^26, 27, 28^

Here we demonstrate that USP2a modulates the susceptibility to antineoplastic agents in prostate cells. In particular, when overexpressed, USP2a^WT^ is able to induce chemo-resistance to typically pro-oxidant agents, such as CDDP and Doxo, and also to the DTX taxane. The latter is in accordance to that previously reported by our group, showing a marked resistance to paclitaxel displayed by the USP2a^WT^ prostate cells if compared with EV (empty vector) and USP2a^MUT^ transfectants.^11^ In this regard, although taxanes are known to be mainly mitotic inhibitors through disruption of microtubule function, recent data on mitochondrial ROS formation showed that they can trigger a direct mitochondrial damage, induce mitochondrial permeability transition and ROS formation.^29, 30, 31^

Cisplatin in particular has been widely used in prostate chemotherapy, along with mitoxanthrone and DTX.^32^ At molecular level, it triggers apoptosis through oxidative stress, p38 kinase activation and mitochondria impairment in different cancer cell types, including prostate cells.^33, 34, 35, 36^ In our experimental model, we demonstrate for the first time that a specific prostatic DUB, USP2a, protects from drug-induced oxidative stress. The molecular mediator of the USP2a protective function is the glutathione. Through miR-driven c-Myc regulation, USP2a implements the intracellular glutathione content by stimulating its synthesis, thus interfering with the oxidative cascade triggered by the chemotherapeutic agents.

## Results

### USP2a overexpression confers resistance to pro-oxidant agents to prostate cells

In a previously characterized experimental model of human immortalized non-transformed prostate epithelial cells (iPrEC), overexpressing either wild-type (USP2a^WT^) or catalytically mutant USP2a (USP2a^MUT^),^11, 19^ we evaluated the response to antineoplastic agents in terms of both subG_1_ percentage and apoptotic PARP cleavage.

**Figure 1:**
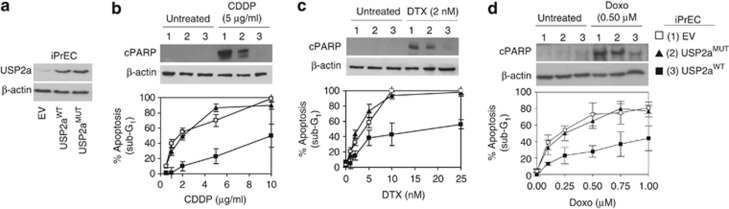
USP2a triggers chemo-resistance in prostate epithelial cells. Evaluation of (**a**) USP2a expression and (**b–d**) of the apoptotic response carried out by western blot of cleaved PARP (cPARP) and cytofluorimetric assay of the subG_1_ percentage in the indicated iPrEC cells, following treatment with CDDP, DTX and Doxo

Figure 1 shows that the ectopic USP2a^WT^ expression (Figure 1a) significantly affects the response to different drugs. Specifically, USP2a^WT^ confers resistance to typically pro-oxidant agents, such as cisplatin (Figure 1b) and doxorubicin (Figure 1d), and to taxanes (docetaxel, Figure 1c, and Priolo *et al.*^11^). The USP2a antiapoptotic function seems to be related to its enzymatic activity, as the dose-dependent effects reported for USP2a^MUT^ resemble those elicited by the empty vector clone. Also in a model of transformed prostate cells (LNCaP cancer cell line), cells acquire resistance to CDDP, DTX and Doxo upon ectopic expression of USP2a^WT^ (Supplementary Figure 1a) as the percentage of both apoptotic cells and PARP cleavage levels is significantly reduced in USP2a^WT^ when compared with control cells (Supplementary Figure 1b). Accordingly, USP2a silencing (Supplementary Figure 1c) triggers a massive increase of apoptosis in response to CDDP, DTX and Doxo treatment, as demonstrated by the augmented subG_1_ percentage and downstream PARP cleavage (Supplementary Figure 1d). Still, the chemo-resistance reported in the LNCaP_USP2a^WT^ cells is specifically dependent on the catalytic activity of the protein, as the drug response observed in the USP2a^MUT^ cells resembles what reported in the LNCaP_EV cells (Supplementary Figure 1e).

### USP2a modulates the cell redox state

**Figure 2:**
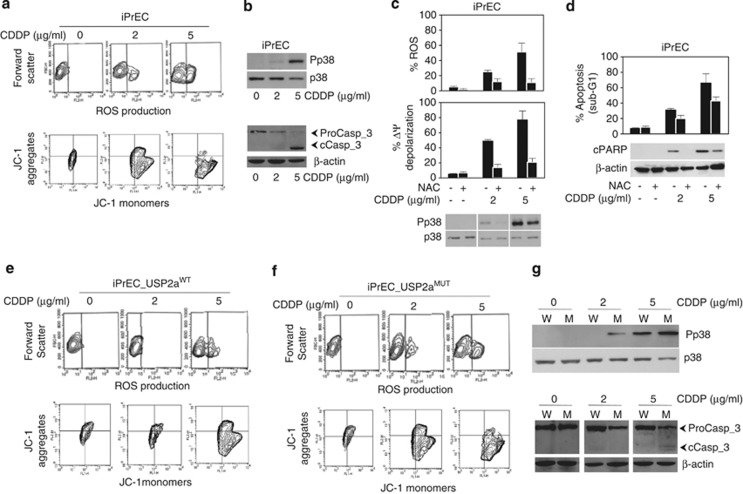
USP2a has an antioxidant effect in response to CDDP. Evaluation of (**a**) ROS production and ΔΨ depolarization, and (**b**) p38 kinase phosphorylation (Pp38) and caspase-3 activation, carried out in iPrEC cells, following CDDP exposure. Upon NAC pre-treatment, iPrEC cells have been characterized in response to CDDP in terms of (**c**) ROS production, ΔΨ depolarization and p38 activation, and of (**d**) cPARP cleavage and subG_1_ apoptotic percentage. Evaluation of (**e**, **f**) ROS and ΔΨ depolarization, and (**g**) p38 and casapse-3 activation has been further carried out in iPrEC_USP2a^WT^ (W) and iPrEC_USP2a^MUT^ (M) cells, respectively, following CDDP exposure

Cisplatin is known to trigger apoptosis through oxidative stress, p38 kinase activation and mitochondria impairment in different cancer cell types, including prostate.^34, 35, 36^ We thus evaluated the generation of ROS and the depolarization of mitochondria membrane potential in iPrEC cells, following CDDP administration. Cytofluorimetric analysis revealed that the susceptibility of epithelial prostate cells to CDDP proceeds via a dose-dependent ROS generation and ΔΨ depolarization (Figure 2a), accompanied by activation of p38 through phosphorylation and caspase-3 cleavage (Figure 2b). CDDP-induced oxidative stress appears to be an upstream molecular event, as specific inhibition of ROS generation by NAC (*N*-acetyl-cysteine) pre-treatment (Figure 2c) reduces p38 phosphorylation, mitochondria depolarization and downstream apoptosis, as evidenced by decreased PARP cleavage and subG_1_ percentage (Figure 2d). However, as NAC pre-loading does not completely abolish p38 activation and PARP cleavage at the CDDP concentration of 5 *μ*g/ml (Figures 2c and d), it suggests that the drug-induced apoptosis can involve alternative pathways at the higher doses. As detailed in Supplementary Figure 2, only when administered in combination with the specific p38 inhibitor (SB202190), NAC interferes with the caspase-3 and PARP activation (Supplementary Figure 2a) and reduces cell death (Supplementary Figure 2b), suggesting the occurrence of a double lane of cellular mediators (both ROS and p38) to death (Supplementary Figure 2c).

Interestingly, when overexpressed, USP2a^WT^ displays an antioxidant effect. Its expression reduces the CDDP-generated ROS and protects prostate cells from drug-induced ΔΨ impairment at both the doses administered (Figure 2e). If catalytically inactive, USP2a does not modify the iPrEC sensitivity to CDDP-triggered oxidative stress and mitochondria dysfunction, the iPrEC-USP2a^MUT^ profile being comparable to control cells (Figure 2f). Still, the ROS-scavenging ability driven by USP2a^WT^ in response to CDDP is able to inhibit downstream p38 phosphorylation and caspase-3 cleavage if compared with mutant protein (Figure 2g). No difference in p38 phosphorylation has been reported between mutant and empty vector cells in response to drug (data not shown).

Also in response to Doxo and DTX, iPrEC cells display an increased ROS generation, which is actively scavenged by NAC pre-exposure (Supplementary Figure 3a). Interestingly, NAC treatment abolishes Doxo-induced citotoxicity, whereas it only partially interferes with the apoptotic cascade initiated by DTX (Supplementary Figure 3b). As USP2a^WT^ scavenges drug-induced ROS species, as much as NAC pre-treatment (Supplementary Figure 3c), but also significantly impairs apoptotic response to DTX (Figure 1c), it suggests that the USP2a-dependent chemo-resistance to taxanes proceeds via multiple pathways and that its antioxidant function is just one of the involved mechanism.

**Figure 3:**
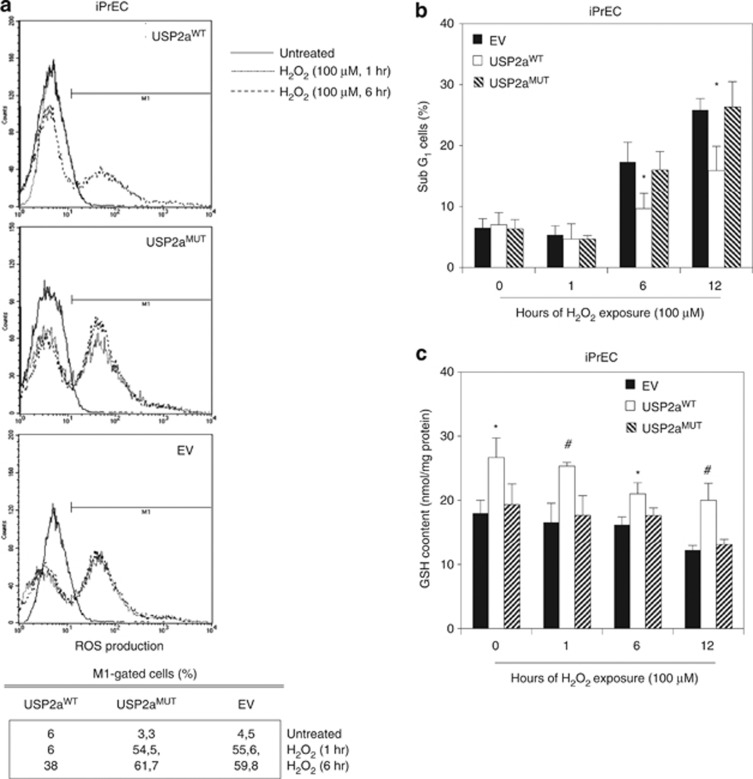
USP2a increases GSH content and scavenges ROS. (**a**) Analysis of ROS production performed in the iPrEC cells exposed to 100 *μ*M H_2_O_2_, for 1 and 6 h. In the table below, the percentage of the M_1_-gated (ROS positive) cells is reported. (**b**) Apoptotic evaluation and (**c**) GSH intracellular content analysis performed in iPrEC cells treated with 100 *μ*M H_2_O_2_ up to 12 h. Asterisks indicate the statistically significant differences, calculated in iPrEC_USP2a^WT^ *versus* EV (*P<0.05, ^#^*P*<0.01)

To further characterize the antioxidant function, USP2a^WT^ overexpression was tested in response to direct H_2_O_2_ exposure in iPrEC cells (Figure 3). As hypothesized, it protects from H_2_O_2_-induced ROS in a time-dependent manner (Figure 3a), the M_1_-gated cells percentage (ROS-positive cells) being significantly reduced in USP2a^WT^ compared with USP2a^MUT^ and empty vector cells, at both 1 and 6 h post treatment. By extending the time-window of the analysis up to 12 h, USP2a^WT^ cells are further demonstrated to be protected from the H_2_O_2_-driven cytotoxic effect (Figure 3b). As the main master regulator of cellular redox homeostasis is the glutathione (GSH), we measured the intracellular GSH content in iPrEC transfectants (Figure 3c) and showed that USP2a^WT^ buffers the H_2_O_2_-induced oxidative stress by implementing the GSH pool in prostate cells. In iPrEC-USP2a^WT^ cells, GSH content is indeed increased by almost 30% if compared with USP2a^MUT^ (Figure 3c) and empty vector control clone.

In support of these findings, deregulated USP2a expression is able to significantly affect GSH concentration also in the LNCaP experimental model (Supplementary Figure 4a).

### miR-dependent c-Myc regulation is involved in the USP2a antioxidant ability

In search of a molecular effector underlying the USP2a antioxidant ability, we evaluated the involvement of c-Myc oncoprotein in the USP2a chemo-protection, as it is a well-known drug resistance factor in cancer,^34, 37, 38^ and is upregulated by USP2a via miR-34b/c impairment in prostate cells.^19^

**Figure 4:**
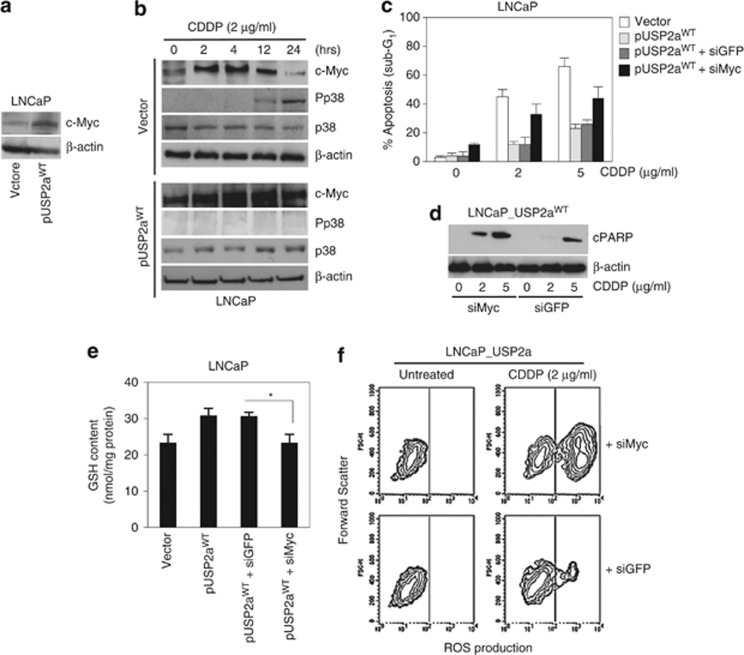
c-Myc is the downstream mediator of the USP2a antioxidant ability in response to drugs. (**a**, **b**) Western blot analysis of c-Myc, basal and phosphorylated p38 (Pp38) expressions carried out in LNCaP empty vector and pUSP2a^WT^ cells, following treatment with CDDP (2 *μ*g/ml). (**c**) Cytofluorimetric evaluation of apoptotic subG_1_ percentage and (**d**) cleaved PARP (cPARP) performed in the following CDDP-treated LNCaP transfectants: empty vector, pUSP2a^WT^,GFP-silenced (siGFP) and c-Myc-silenced (siMyc) pUSP2a^WT^ cells. Evaluation of (**e**) GSH content and (**f**) CDDP-induced ROS production carried out in the indicated LNCaP_USP2a^WT^ transfectants. Asterisks indicate the statistically significant differences, calculated in siMyc *versus* siGFP LNCaP_USP2a^WT^ transfectants (*P<0.05)

To this purpose, we first verified whether c-Myc expression was induced by drug treatment in the USP2a^WT^ and control LNCaP cells (Figures 4a and b). In response to CDDP, LNCaP cells attempt to react to drug-induced damage by increasing c-Myc protein as early as 2 h after drug administration (Figure 4b). c-Myc expression level is maintained for the following 12 h, returning progressively to basal level by 24 h. Interestingly, activation of p38 is inversely correlated to c-Myc induction, the kinase being progressively phosphorylated with the disappearance of c-Myc. Through upregulation of basal c-Myc level (Figure 4a), USP2a^WT^ confers resistance to CDDP by enabling LNCaP cells to quickly respond to drug damage. In fact, c-Myc protein expression is kept higher than in empty vector transfectants, and no phosphorylation of p38 kinase can be triggered by drug in USP2a^WT^ cells. The ability of c-Myc to specifically protect cells from CDDP-induced damage was confirmed by evaluating the drug response in c-Myc-overexpressing prostate cancer cells. c-Myc stimulation in LNCaP cells (Supplementary Figure 5a) abrogates CDDP-induced p38 phosphorylation (Supplementary Figure 5b), reduces the apoptotic subG_1_ percentage in a dose-dependent manner, even more efficiently than what reported for USP2a^WT^ overexpression (Supplementary Figure 5c), and inhibits PARP cleavage (Supplementary Figure 5d). In accordance, c-Myc interference in LNCaP_USP2a^WT^-overexpressing cells partially reverts CDDP resistance. As shown in Figure 4c, siMyc stimulates apoptosis in response to cisplatin and leads to a concomitant appearance of cleaved PARP at the lowest dose reported (Figure 4d), although the drug susceptibility is never completely restored to parental level.

c-Myc-driven protection from CDDP in USP2a^WT^ cells proceeds via GSH modulation. As reported in Figure 4e, siMyc depletes GSH content in the LNCaP_USP2a^WT^, down to basal levels, thus interfering with the ROS scavenging properties gained with USP2a^WT^ overexpression (Figure 4f). We previously demonstrated that c-Myc transcription factor regulates the *γ*-glutamyl-cysteine synthetase (*γ*-GCS), the rate-limiting enzyme catalyzing GSH biosynthesis, by directly binding and activating the promoters of both heavy (*γ*-GCS_H_) and light (*γ*-GCS_L_) subunits.^34, 39^ We therefore verified whether the GSH modulation may proceed via a Myc-dependent regulation of *γ*-GCS expression in our USP2a^WT^ prostate experimental model. As reported in Supplementary Figure 6, USP2a^WT^ stimulates both *γ*-GCS subunit expression in LNCaP cells, in terms of mRNA, protein, and enzyme activity rise. Consistently, siUSP2a-LNCaP cells are characterized by a significant decrease in *γ*-GCS activity (Supplementary Figure 4b). This expression control is clearly USP2a^WT^-specific, as both LNCaP_USP2a^MUT^ and iPrEC_USP2a^MUT^ cells resemble what is observed in the empty vector clones (Supplementary Figure 4b).

As hypothesized, c-Myc is the master regulator in the USP2a-driven *γ*-GCS regulation, as c-Myc silencing reverts the expression and activity stimulation elicited by USP2a^WT^ (Supplementary Figures 6a–d).

**Figure 5:**
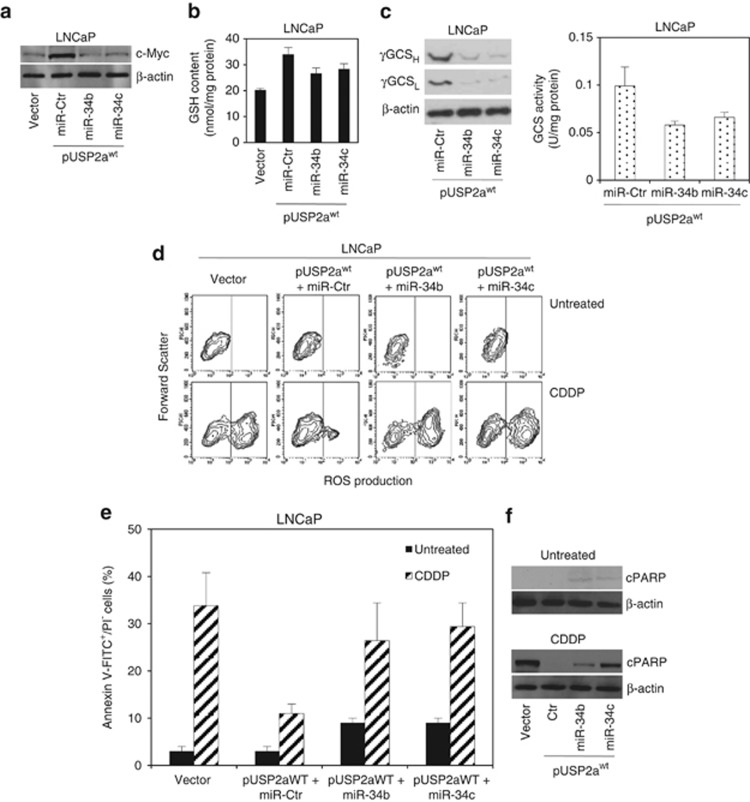
miR-34b/c are involved in the GSH-mediated chemo-resistance driven by USP2a. (**a**) Western blot analysis of c-Myc. (**b**) GSH content evaluation, and (**c**) analysis of the *γ*-GCS protein expression and enzyme activity carried out in LNCaP empty vector and pUSP2a^WT^ transfectant. The latter cells have also been incubated with control miR mimic (miR-Ctr), miR-34b and miR-34c mimic molecules. Analysis of (**d**) ROS generation, (**e**) apoptosis (Annexin V/PI staining) and (**f**) PARP cleavage (cPARP) performed in the above detailed miR mimic-incubated pUSP2a^WT^ transfectants, following CDDP exposure

We also demonstrated that c-Myc regulation by USP2a occurs via miR-34b/c inhibition.^19^ We next moved to verify whether GSH-dependent drug resistance to pro-oxidant agents might also be reverted by the synthetic miR molecules. To this end, LNCaP_USP2a^WT^ cells have been preloaded with either control or mimic 34b and 34c molecules before undergoing drug treatment (Figure 5). As expected, both miR-34b and miR-34c are able to reduce c-Myc protein expression in prostate cells, whereas the control mimic does not modify its level (Figure 5a). Interestingly, exogenous miR-34b/c loading does not completely counteract c-Myc induction upon CDDP treatment (Supplementary Figure 7), suggesting the occurrence of alternative pathways controlling Myc induction and stabilization by the drug-induced stress.

The glutathione pool undergoes a significant depletion upon mimic miR addition (Figure 5b), and it is in turn responsible for the marked reduced ROS scavenging ability observed in CDDP-treated LNCaP_USP2a^WT^ cells (Figure 5d). miR-dependent GSH depletion is triggered by the impairment of the *γ*-GCS expression and enzyme activity (Figure 5c) in the USP2a^WT^ cells. On the basis of an *in silico* prediction, carried out by interrogating the public databases and algorithms available on the web (Sanger miRbase, PicTar, Target Scan, miRanda, DIANA miRGen, miRNA Map),^31^ we excluded that the inhibition of *γ*-GCS expression in our experimental model is due to a direct targeting by miR-34b/c (data not shown).

The combination of USP2a^WT^ and control mimic molecule still protects cells from CDDP-induced apoptosis, if compared with control clone, as evidenced by the percentage of cells exposing the early Annexin V marker (Figure 5d), and PARP cleavage (Figure 5e). In contrast, pre-treatment with either miR-34b or miR-34c makes USP2a^WT^ cells unable to trigger drug resistance. Actually, miR analogs *per se* are responsible for activating a very small amount of apoptosis in proliferating USP2a^WT^ cells, as demonstrated by the slight increase in Annexin V^+^/propidium iodide (PI)^−^ percentage and by the faint appearance of PARP cleavage in the untreated cells (Figures 5d and e). Moreover, each specific miR analog abolishes the protective effect elicited by USP2a^WT^ in response to CDDP, the percentage of Annexin V^+^/PI^−^ cells being comparable to vector-transfected LNCaP cells (Figure 5d). Analysis of PARP cleavage confirms that both miR-34b and miR-34c are able to revert cisplatin resistance, although at different extent, the relative protein amount being slightly different from parental CDDP-treated cells (Figure 5e).

## Discussion

Despite the critical role of the ubiquitin–proteasome pathway in the regulation of cellular processes and cancer transformation,^3, 40, 41^ surprisingly little is known about the function specificity of individual DUB enzymes. We have recently identified a novel molecular pathway of oncogenic regulation driven by the USP2a deubiquitinating enzyme through its ability to recruit specific miRNAs in prostate cells.^19^ Here we demonstrate that USP2a also triggers prostate chemo-resistance by acting as an antioxidant molecule via the miR-34-mediated Myc and GSH stimulation. To our knowledge, this is the first report disclosing the cooperation between a DUB enzyme and the miR regulatory machinery specifically in response to oxidative stress.

We here report that the ectopic overexpression of USP2a^WT^ drives chemo-resistance in both iPrEC and prostate cancer LNCaP cells, in accordance to that previously suggested in prostate histotype by our group.^11^ This oncogenic property also applies to other cancer cell lines, as seen in bladder cancer and in testicular embryonal carcinoma cells, where USP2a deregulation affects response to cisplatin.^14, 42^ These findings are suggestive of a possible clinical application of USP2a inhibitors to cisplatin-resistant patient population to improve clinical benefit.

Interestingly, in our prostate experimental model, USP2a is able to specifically impair the death response to the pro-oxidant agents, such as CDDP and Doxo, and to microtubules polymerization inhibitors, such as DTX and paclitaxel.^11^ In accordance to our findings, siUSP2a bladder cancer cells are reported to display a higher apoptotic rate in response to CDDP and nocodazole, an antineoplastic agent specifically interfering with the polymerization of microtubules.^42^

This USP2a function can be attributable to its deubiquitinating activity, as the response elicited by the catalytically inactive mutant (C276A and H549R) resembles that reported in control cells. Thus, USP2a might specifically address the chemo-response by deubiquitinating and stabilizing different proteins according to the nature of the stressor. Besides, its action may depend on the cellular balance with other death-regulating effectors. This is particularly evident following the recent publication by Mahul-Mellier on Hela and MCF7 breast cancer cells, where downregulation of USP2a by siRNA promotes NF-kB activation and protects cells against TNF-induced cell death, as a consequence of the impairment of the USP2a-TRAF protein ratio.^43, 44^

Cisplatin has been extensively used in prostate cancer therapy, and, at molecular level, it is known to trigger apoptosis through mitochondria-mediated oxidative stress.^35, 36^ The cytotoxicity of anthracyclines is also associated with the generation of reactive oxygen species (ROS) and reactive nitrogen species (RNS). Besides, Doxo can bind to the DNA and uncoil the double-stranded helix with generation of free radicals and DNA damage.^45, 46^ Interestingly, although taxanes are mainly mitotic inhibitors, through disruption of microtubule function, recent data on mitochondrial ROS formation showed that they can display direct mitochondrial effect, induce mitochondrial permeability transition and ROS formation.^29, 30, 31^

We here demonstrate that ROS generation is a key upstream event responsible for CDDP cytotoxicity in prostate, and that USP2a behaves as a ROS scavenging molecule, even if p38 phosphorylation may occur independently of oxidative stress at higher doses. Both Doxo and DTX also elicit ROS generation, that is efficiently scavenged by USP2a^WT^, although, according to our data, chemo-resistance to taxanes proceeds via multiple pathways, and USP2a antioxidant function is just one of the involved mechanism. On the basis of what already published, resistance to taxanes might be explained in terms of the proteins stabilized by USP2a-deubiquitinating function, especially those involved in apoptosis, such as FASN, Mdm2, MdmX and AIF, or by the multiple miR molecules regulated in the prostate experimental model. Future intensive experiments are required to exhaustively address these issues.

Pro-oxidant chemo-therapy appears particularly efficient in prostate cells. Over the last decade, epidemiological, experimental and clinical studies have in fact implicated oxidative stress in the development and progression of prostate cancer.^47^ A possible explanation identifies in the oxidative stress the key factor responsible for the activation of androgen receptor signaling and for the pro-survival and antiapoptotic effects in response to androgen-deprivation therapy, as well as to cytotoxic and tumor-suppressive interventions.^48^

In the light of these evidences, it is comprehensible that the impact of USP2a overexpression in a prostate lesion, as, in response to CDDP, it displays an antioxidant effect. To our knowledge, this is the first experimental evidence demonstrating a ROS-scavenging role for USP2a and, more important, identifying in the intracellular GSH neo-synthesis the possible mediator of this protective effect. This finding is surprisingly in accordance to what has recently been published by Selvendiran *et al.*^49^ They report that the synthetic compound HO-3867, known for its potent antioxidant activity, exhibits anticancer effects in many histotypes, including the breast, colon, head and neck, liver, lung, ovarian and prostate, by downregulating USP2a to ultimately trigger apoptosis.^49^

There is an emerging evidence supporting the role of miRNAs in modulating sensitivity to anticancer therapy, and miR-34a and miR-34b/c family in particular has been associated to prostate cancer survival and drug sensitivity.^50, 51, 52, 53^ We recently demonstrated that USP2a switches on Myc expression via miR-34b/c regulation.^19^ The ability to affect c-Myc level by miR has its functional relevance in the regulation of apoptosis and response to chemotherapy. c-Myc is in fact known to be a drug resistance factor.^34, 37, 38^ Different c-Myc-targeted antisense therapies have been so far developed and tested as reliable strategies to improve chemotherapy in solid tumors, strongly identifying in c-Myc a key factor of drug resistance and stress response. In prostate, *c-myc* is often amplified in advanced cancer specimens^54^, so that targeted inhibition of its overexpression has been demonstrated as a promising therapeutic strategy for clinical management of prostate cancer.^55^ Moreover, therapeutic use of *c-myc* antisense renders hormone-refractory prostate cancers responsive to chemotherapy in the preclinical model.^56^

Here we demonstrate that USP2a^WT^ confers drug resistance by upregulating c-Myc expression and by implementing the GSH content. This is directly due to the ability of c-Myc transcription factor to stimulate the expression of *γ*-GCS, the rate-limiting enzyme catalyzing the GSH biosynthesis. Moreover, miR-34b/c-mediated c-Myc stimulation is set upstream the GSH neo-synthesis, as both miR mimic administration and Myc silencing in LNCaP-USP2a^WT^ cells are responsible for *γ*-GCS inhibition and consequent GSH depletion, with further chemo-sensitization to pro-oxidant agents. This is consistent with our previously reported data demonstrating a direct antioxidant role of c-Myc through regulation of glutathione synthesis,^34, 39^ also corroborated by Gao *et al* who disclosed c-Myc involvement in energy and reactive oxygen species homeostasis.^57^ These data might strengthen the hypothesis of a therapeutic approach in USP2a-overexpressing prostate lesions based on the combination of conventional chemotherapy with either miR synthetic drugs or siMyc.

**Figure 6:**
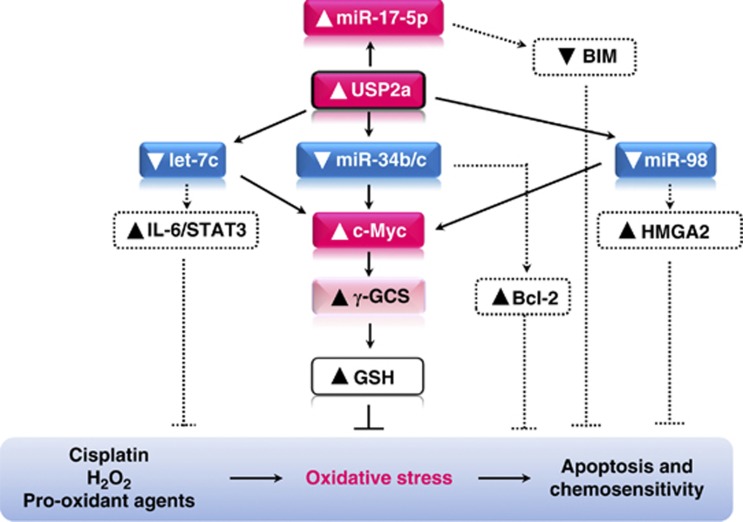
USP2a-activated pathways in response to oxidative stress. The schematized molecular model describes the multiple miRNA-target connections triggered by USP2a overexpression in prostate cells, in response to pro-oxidant agents. Solid lines represent that demonstrated by our present and recently published data.^19^ Dotted lines suggest further potential USP2a-miRNA-target cross-talks, underlying the antioxidant and chemo-protective function elicited by USP2a in prostate

On the basis of our data, we propose a molecular model where USP2a is the upstream master regulator, driving the response to ROS-generating drugs via miR-34b/c-Myc-GSH in prostate cells (Figure 6). In the scheme, we also highlight those miRNAs that we previously identified as USP2a targets, such as let-7, miR-98 and miR-17-5p.^19^ According to published evidence, they are known to interfere with the expression of specific targets (BIM, IL-6/STAT3, HMGA2 and Bcl-2), specifically involved in the apoptotic response to pro-oxidant agents and antineoplastic drugs.^58, 59, 60^ In our hypothesis, prostate chemo-resistance might be regulated by USP2a through multiple pathways, according to the dose and the nature of the stressor. Further studies in this direction will help in better defining the involvement of each specific molecular mediator in the complex web of interaction woven by USP2a in prostate cancer cells.

## Materials and Methods

### Chemicals, antibodies and plasmids

Clinical-grade cisplatin and doxorubicin were obtained from Pharmacia (Milan, Italy). Docetaxel was purchased from Sigma (Sigma-Aldrich, St. Louis, MO, USA) and Glaxo Wellcome (Verona, Italy), respectively. Drug dilutions were freshly prepared before each experiment.

NAC, H_2_O_2_ (30% w/w) and PI were from Sigma. DHE (dihydroethidium), JC-1 (5′,6,6′-tetrachloro-1,1′,3,3′-tetraethylbenzimidazolylcarbocyanine iodide) and the Annexin V-FITC *versus* PI kit (Vibrant apoptosis assay, V-13242) were purchased from Molecular Probes (Life Technologies, Grand Island, NY, USA).

For western blot analysis, the following primary antibodies have been used: anti-USP2a (N-term and C-term, Abgent, San Diego, CA, USA), anti-c-Myc (clone 9E10, Santa Cruz, CA, USA), anti-procaspase-3 and cleaved caspase-3 (Upstate Biotechnology, New York, NY), anti-cleaved PARP (cPARP, Roche Applied Sciences, Indianapolis, IN, USA), anti-p38 and phospoho-p38 (Cell Signaling Technology, Danvers, MA, USA), anti-*γ*GCS_H_ and anti-*γ*GCS_L_ (Santa Cruz), anti-GAPDH (6C5, Santa Cruz) and anti-beta actin (AC-74, Sigma).

Overexpression experiments have been performed by transfecting cells with pCDNA3 empty vector (Invitrogen, Life Technologies), pCDNA3-USP2a^WT^ (pUSP2a^WT^) and pCDNA3-USP2a^MUT^ (pUSP2a^MUT^), as previously described.^11, 19^ Silencing oligonucleotides (siRNA) for knockdown experiments have been purchased from MWG (Huntsville, AL, USA), as previously detailed.^19^ Synthetic (Mimic) miRNA sequences (control, miR-34b and 34c) were purchased from Dharmacon (Thermo Scientific, Pittsburgh PA, USA). Lyophilized molecules have been dissolved in diethylpyrocarbonate (DEPC) water, stocked and freshly diluted before experiments, according to the manufacturer's instructions.

### Cell culture and transfection

Empty vector (Vector), wild-type (USP2a^WT^) and mutant (USP2a^MUT^) stable clones (previously established by infecting immortalized androgen receptor-expressing prostate epithelial cells (iPrEC)^11, 19^ were grown in specific PrEBM medium (Cambrex, East Rutherford, NJ, USA) and selected in 1.6 *μ*g/ml puromycin. Human prostate adenocarcinoma LNCaP cells were obtained from the American Type Culture Collection (ATCC, Manassas, VA, USA) and grown in RPMI-1640 medium (Invitrogen) containing 10% fetal bovine serum (GIBCO-Invitrogen) and 1% penicillin-streptomycin (Invitrogen).

Transient transfection experiments were carried out by seeding cells in 60-mm Petri dishes in complete medium (2 × 10^5^ cells/plate). Transfection was performed 24 h after plating using Lipofectamine 2000 reagent (Invitrogen) in Optimem medium (Invitrogen) for siRNA oligonucleotides (100–500 nM) and synthetiC miR molecules (10 nM), and by JetPei reagent (PloyPlus-Transfection, New York, NY, USA) in complete medium for expression vectors (0.5–5 *μ*g). Evaluation of protein, mRNA and miRNA expression levels was performed by harvesting cells at 24 h intervals (24–120 h) after transfection.

### Treatment with antineoplastic agents and H_2_O_2_

For drug treatment, cells were seeded in 60-mm Petri dishes at a density of 2 × 10^5^ cells/plate. After 24 h, cells were exposed for 2–24 h to different doses of CDDP (ranging from 0.5 to 10 *μ*g/ml), DTX (ranging from 1 to 25 nM) and Doxo (ranging from 0.1 to 1 *μ*M). In the experiments with NAC antioxidant, cells were incubated with 5 mM NAC (dose with no toxic effect on cell survival) 6 h before drug administration.

In the experiments with H_2_O_2_, iPrEC cells (2 × 10^5^ cells/plate) were incubated with 100 *μ*M of the pro-oxidant agent for 1–12 h and immediately analyzed for apoptosis, GSH content and ROS generation.

### Western blot

Cell lysis was performed on ice for 30 min in RIPA buffer (50 mM Tris-HCl pH 7.4, 150 mM NaCl, 1% NP-40, 0.25% sodium deoxycholate, 1 mM EDTA) supplemented with phosphatase inhibitors (1 mM PMSF, 1 *μ*g/ml aprotinin, leupeptin, pepstatin). Equal amounts of total protein extracts (20–80 μg) were resolved by 10%, 12% or 15% denaturing SDS polyacrylamide gel electrophoresis (SDS–PAGE) and transferred for 4 h to the polyvinylidene difluoride membrane. Membranes were blocked in 5% milk-PBS-0.05% Tween 20 for 1 h and incubated overnight with the specific primary antibodies. Secondary antibodies were horseradish peroxidase-conjugated (Santa Cruz), and ECL reagent (Amersham, GE Healthcare, Piscataway, NJ, USA) was employed for chemo-luminescence detection.

### GSH Determination

Intracellular GSH content was measured as previously described^19^, using a colorimetric assay (Bioxytech GSH-400; Oxis International Inc., Beverly Hills, CA, USA), according to the manufacturer's instruction.

### *γ*GCS enzyme activity

Enzymatic activity was evaluated by using a coupled assay with pyruvate kinase and lactate dehydrogenase and assaying the rate of decrease in absorbance at 340 nm at 37 °C. Enzyme activity was expressed as mmol of NADH oxidized per minute (U) per mg protein.^34^

### Flow cytometric evaluation of apoptosis

Apoptosis was detected by both PI staining of 80% ethanol-fixed cells and Annexin V-FITC *versus* PI assay performed in fresh cells, as previously described.^19^

Briefly, ethanol-fixed cells were washed in phosphate buffered saline (PBS), stained in a solution (containing RNase A (50 *μ*g/ml, Sigma), Triton 0.1%, EDTA (0.1 mM, Sigma) and PI (50 *μ*g/ml) in PBS) and incubated in the dark for at least 30 min. SubG_1_ percentage has been determined by analyzing DNA content using the flow cytometer (Beckton and Dickinson, Frankin Lakes, NJ, USA). For the Annexin assay, adherent cells were harvested, suspended in the annexin-binding buffer (about 1 × 10^6^ cells/ml) and incubated with the Annexin V-FITC and PI for 15 min, at room temperature in the dark, then immediately analyzed using flow cytometry. These data are presented as bi-parametric dot plots showing the annexin V-FITC green fluorescence *versus* the PI red fluorescence. Analysis of cell death was performed from 0 to 96 h after the end of each drug treatment.

### Assessment of the mitochondrial membrane potential

ΔΨ was assayed by staining cells with the JC-1 probe, a cationic dye that exhibits mitochondria potential-dependent accumulation, without being affected by the plasmalemma potential. JC-1 accumulates in the cytoplasm, where it produces green fluorescence and forms red fluorescent J-aggregates in the mitochondria. Mitochondria depolarization is indicated by a decrease in the red/green fluorescence intensity ratio. Adherent cells (about 5 × 10^5^) were first assayed for viability and then loaded with 10 mM JC-1 in complete medium, for 30 min at 37 °C in the dark. After incubation, cells were washed twice and suspended in PBS, then immediately analyzed using flow cytometry (Beckton and Dickinson). These data are presented as bi-parametric panels with the green J-monomers fluorescence plotted *versus* the red J-aggregates fluorescence. Analysis of ΔΨ was performed from 0 to 72 h following the end of drug administration.

### Evaluation of the reactive oxygen species

The evaluation of ROS production was performed as previously described.^19^ Briefly, adherent cells (about 5 × 10^5^) were first assayed for viability and then incubated with 4 mM DHE for 45 min at 37 °C in PBS. After incubation, cells were immediately analyzed using flow cytometry. These data are presented as both mono-parametric histograms or bi-parametric panels with the red DHE fluorescence intensity plotted *versus* the forward scatter. Analysis of ROS generation was performed from 0 to 72 h following the end of each drug treatment.

### Statistical analysis

All data are presented as mean±S.D., calculated in ⩾ three replicates. Statistical analyses were performed using an unpaired two-tailed *t* test using SPSS Software Version One. *P*-values <0.05 were taken as statistically significant.

## Abbreviations

CDDP: cisplatin
CPT: camptothecin
ΔΨ: mitochondria membrane potential
Doxo: doxorubicin
DUB: deubiquitinating enzyme
DTX: docetaxel
EV: empty vector
γGCS: the γ-glutamyl-cysteine synthetase
GSH: glutathione
iPrEC: human immortalized non-transformed prostate epithelial cells
miRNAs or miRs: microRNAs
NAC: *N*-acetyl-cysteine
PI: propidium iodide
ROS: reactive oxygen species
USP2a: ubiquitin-specific protease 2a
USP2aWT: wild-type ubiquitin-specific protease 2a
USP2aMUT: mutant (C276A and H549R) ubiquitin-specific protease 2a

## References

[bib1] Bernards R A genomic and functional inventory of deubiquitinating enzymes Cell 2005 123 773 786 16325574 10.1016/j.cell.2005.11.007

[bib2] Salmena L Pandolfi PP Changing venues for tumour suppression: balancing destruction and localization by monoubiquitylation Nat Rev Cancer 2007 7 409 413 17508027 10.1038/nrc2145

[bib3] Yang Y Kitagaki J Wang H Hou DX Perantoni AO Targeting the ubiquitin-proteasome system for cancer therapy Cancer Sci 2009 100 24 28 19037995 10.1111/j.1349-7006.2008.01013.x PMC2643214

[bib4] Ruschak AM Slassi M Kay LE Schimmer AD Novel proteasome inhibitors to overcome bortezomib resistance J Natl Cancer Inst 2011 103 1007 1017 21606441 10.1093/jnci/djr160

[bib5] Inuzuka H Fukushima H Shaik S Liu P Lau AW Wei W Mcl-1 ubiquitination and destruction Oncotarget 2011 2 239 244 21608150 10.18632/oncotarget.242 PMC3260810

[bib6] Neznanov N Komarov AP Neznanova L Stanhope-Baker P Gudkov AV Proteotoxic stress targeted therapy (PSTT): induction of protein misfolding enhances the antitumor effect of the proteasome inhibitor bortezomib Oncotarget 2011 2 209 221 21444945 10.18632/oncotarget.246 PMC3260823

[bib7] Donna LD Lagadec C Pajonk F Radioresistance of prostate cancer cells with low proteasome activity Prostate 2011 72 868 874 21932424 10.1002/pros.21489 PMC3396561

[bib8] Jandial DD Farshchi-Heydari S Larson CA Elliott GI Wrasidlo WJ Howell SB Enhanced delivery of cisplatin to intraperitoneal ovarian carcinomas mediated by the effects of bortezomib on the human copper transporter 1 Clin Cancer Res 2009 15 553 560 19147760 10.1158/1078-0432.CCR-08-2081 PMC2707998

[bib9] Liu L Yang C Herzog C Seth R Kaushal GP Proteasome inhibitors prevent cisplatin-induced mitochondrial release of apoptosis-inducing factor and markedly ameliorate cisplatin nephrotoxicity Biochem Pharmacol 2010 79 137 146 19699182 10.1016/j.bcp.2009.08.015

[bib10] Stringer DK Piper RC Terminating protein ubiquitination: Hasta la vista, ubiquitin Cell Cycle 2011 10 3067 3071 21926471 10.4161/cc.10.18.17191 PMC3685619

[bib11] Priolo C Tang D Brahamandan M Benassi B Sicinska E Ogino S The isopeptidase USP2a protects human prostate cancer from apoptosis Cancer Res 2006 66 8625 8632 16951176 10.1158/0008-5472.CAN-06-1374

[bib12] Graner E Tang D Rossi S Baron A Migita T Weinstein LJ The isopeptidase USP2a regulates the stability of fatty acid synthase in prostate cancer Cancer Cell 2004 5 253 261 15050917 10.1016/s1535-6108(04)00055-8

[bib13] Stevenson LF Sparks A Allende-Vega N Xirodimas DP Lane DP Saville MK The deubiquitinating enzyme USP2a regulates the p53 pathway by targeting Mdm2 EMBO J 2007 26 976 986 17290220 10.1038/sj.emboj.7601567 PMC1852834

[bib14] Allende-Vega N Sparks A Lane DP Saville MK MdmX is a substrate for the deubiquitinating enzyme USP2a Oncogene 2010 29 432 441 19838211 10.1038/onc.2009.330

[bib15] Oh KH Yang SW Park JM Seol JH Iemura S Natsume T Control of AIF-mediated cell death by antagonistic functions of CHIP ubiquitin E3 ligase and USP2 deubiquitinating enzyme Cell Death Differ 2011 18 1326 1336 21293491 10.1038/cdd.2011.3 PMC3172096

[bib16] Shi Y Solomon LR Pereda-Lopez A Giranda VL Luo Y Johnson EF The deubiquitinase USP2A regulates the stability of aurora-A J Biol Chem 2011 286 38960 38968 21890637 10.1074/jbc.M111.231498 PMC3234721

[bib17] Shan J Zhao W Gu W Suppression of cancer cell growth by promoting cyclin D1 degradation Mol Cell 2009 36 469 476 19917254 10.1016/j.molcel.2009.10.018 PMC2856324

[bib18] Liu Z Zanata SM Kim J Peterson MA Di Vizio D Chirieac LR The ubiquitin-specific protease USP2a prevents endocytosis-mediated EGFR degradation Oncogene 2012 32 1660 1669 22710717 10.1038/onc.2012.188 PMC3866888

[bib19] Benassi B Flavin R Marchionni L Zanata S Pan Y Chowdhury D MYC is activated by USP2a-mediated modulation of microRNAs in prostate cancer Cancer Discov 2012 2 236 247 22585994 10.1158/2159-8290.CD-11-0219 PMC3523361

[bib20] Volinia S Calin GA Liu CG Ambs S Cimmino A Petrocca F A microRNA expression signature of human solid tumors defines cancer gene targets Proc Natl Acad Sci USA 2006 103 2257 2261 16461460 10.1073/pnas.0510565103 PMC1413718

[bib21] Porkka KP Pfeiffer MJ Waltering KK Vessella RL Tammela TL Visakorpi T MicroRNA expression profiling in prostate cancer Cancer Res 2007 67 6130 6135 17616669 10.1158/0008-5472.CAN-07-0533

[bib22] Ozen M Creighton CJ Ozdemir M Ittmann M Widespread deregulation of microRNA expression in human prostate cancer Oncogene 2008 27 1788 1793 17891175 10.1038/sj.onc.1210809

[bib23] Hummel R Hussey DJ Haier J MicroRNAs: predictors and modifiers of chemo- and radiotherapy in different tumour types Eur J Cancer 2010 46 298 311 19948396 10.1016/j.ejca.2009.10.027

[bib24] Fujita Y Kojima K Ohhashi R Hamada N Nozawa Y Kitamoto A MiR-148a attenuates paclitaxel resistance of hormone-refractory, drug-resistant prostate cancer PC3 cells by regulating MSK1 expression J Biol Chem 2010 285 19076 19084 20406806 10.1074/jbc.M109.079525 PMC2885186

[bib25] Xu B Niu X Zhang X Tao J Wu D Wang Z miR-143 decreases prostate cancer cells proliferation and migration and enhances their sensitivity to docetaxel through suppression of KRAS Mol Cell Biochem 2011 350 207 213 21197560 10.1007/s11010-010-0700-6

[bib26] Fujita Y Kojima K Hamada N Ohhashi R Akao Y Nozawa Y Effects of miR-34a on cell growth and chemoresistance in prostate cancer PC3 cells Biochem Biophys Res Commun 2008 377 114 119 18834855 10.1016/j.bbrc.2008.09.086

[bib27] Rokhlin OW Scheinker VS Taghiyev AF Bumcrot D Glover RA Cohen MB MicroRNA-34 mediates AR-dependent p53-induced apoptosis in prostate cancer Cancer Biol Ther 2008 7 1288 1296 18497571 10.4161/cbt.7.8.6284

[bib28] Kojima K Fujita Y Nozawa Y Deguchi T Ito M MiR-34a attenuates paclitaxel-resistance of hormone-refractory prostate cancer PC3 cells through direct and indirect mechanisms Prostate 2010 70 1501 1512 20687223 10.1002/pros.21185

[bib29] Varbiro G Veres B Gallyas F Jr Sumegi B Direct effect of taxol on free radical formation and mitochondrial permeability transition Free Radical Biol Med 2001 31 548 558 11498288 10.1016/s0891-5849(01)00616-5

[bib30] Ozben T Oxidative stress and apoptosis: impact on cancer therapy Journal of Pharmacological Science 2007 96 2181 2196 10.1002/jps.20874 17593552

[bib31] Mediavilla-Varela M Pacheco FJ Almaguel F Perez J Sahakian E Daniels TR Docetaxel-induced prostate cancer cell death involves concomitant activation of caspase and lysosomal pathways and is attenuated by LEDGF/p75 Molecular Cancer 2009 28 68 74 19715609 10.1186/1476-4598-8-68 PMC2741463

[bib32] Shah N Dizon DS New-generation platinum agents for solid tumors Future Oncol 2009 5 33 42 19243296 10.2217/14796694.5.1.33

[bib33] Itoh T Terazawa R Kojima K Nakane K Deguchi T Ando M Cisplatin induces production of reactive oxygen species via NADPH oxidase activation in human prostate cancer cells Free Radic Res 2011 45 1033 1039 21682664 10.3109/10715762.2011.591391

[bib34] Benassi B Fanciulli M Fiorentino F Porrello A Chiorino G Loda M c-Myc phosphorylation is required for cellular response to oxidative stress Mol Cell 2006 21 509 519 16483932 10.1016/j.molcel.2006.01.009

[bib35] Schweyer S Soruri A Heintze A Radzun HJ Fayyazi A The role of reactive oxygen species in cisplatin-induced apoptosis in human malignant testicular germ cell lines Int J Oncol 2004 25 1671 1676 15547704 10.3892/ijo.25.6.1671

[bib36] Bragado P Armesilla A Silva A Porras A Apoptosis by cisplatin requires p53 mediated p38alpha MAPK activation through ROS generation Apoptosis 2007 12 1733 1742 17505786 10.1007/s10495-007-0082-8

[bib37] el-Deiry WS Role of oncogenes in resistance and killing by cancer therapeutic agents Curr Opin Oncol 1997 9 79 87 9090498 10.1097/00001622-199701000-00013

[bib38] Hattinger CM Stoico G Michelacci F Pasello M Scionti I Remondini D Mechanisms of gene amplification and evidence of coamplification in drug-resistant human osteosarcoma cell lines Genes Chromosomes Cancer 2009 48 289 309 19105235 10.1002/gcc.20640

[bib39] Benassi B Zupi G Biroccio A Gamma-glutamylcysteine synthetase mediates the c-Myc-dependent response to antineoplastic agents in melanoma cells Mol Pharm 2007 72 1015 1023 10.1124/mol.107.038687 17628013

[bib40] Yang Y Li CC Weissman AM Regulating the p53 system through ubiquitination Oncogene 2004 23 2096 2106 15021897 10.1038/sj.onc.1207411

[bib41] Ruffner H Joazeiro CA Hemmati D Hunter T Verma IM Cancer-predisposing mutations within the RING domain of BRCA1: loss of ubiquitin protein ligase activity and protection from radiation hypersensitivity Proc Natl Acad Sci USA 2001 98 5134 5139 11320250 10.1073/pnas.081068398 PMC33176

[bib42] Kim J Kim WJ Liu Z Loda M Freeman MR The ubiquitin-specific protease USP2a enhances tumor progression by targeting cyclin A1 in bladder cancer Cell Cycle 2012 11 1123 1130 22370483 10.4161/cc.11.6.19550 PMC3335918

[bib43] Mahul-Mellier AL Pazarentzos E Datler C Iwasawa R AbuAli G Lin B Grimm S De-ubiquitinating protease USP2a targets RIP1 and TRAF2 to mediate cell death by TNF Cell Death Differ 2012 19 891 899 22179575 10.1038/cdd.2011.185 PMC3321630

[bib44] Mahul-Mellier AL Datler C Pazarentzos E Lin B Chaisaklert W Abuali G Grimm S De-ubiquitinating proteases USP2a and USP2c cause apoptosis by stabilising RIP1 Biochim Biophys Acta 2012 1823 1353 1365 22659130 10.1016/j.bbamcr.2012.05.022

[bib45] Nicolson GL Conklin KA Reversing mitochondrial dysfunction, fatigue and the adverse effects of chemotherapy of metastatic disease by molecular replacement therapy Clin Exp Metastasis 2008 25 161 169 18058028 10.1007/s10585-007-9129-z

[bib46] Pieniążek A Czepas J Piasecka-Zelga J Gwoździński K Koceva-Chyła A Oxidative stress induced in rat liver by anticancer drugs doxorubicin, paclitaxel and docetaxel Adv Med Sci 2013 20 24 31 10.2478/v10039-012-0063-1 23612702

[bib47] Battisti V Maders LD Bagatini MD Reetz LG Chiesa J Battisti IE Oxidative stress and antioxidant status in prostate cancer patients: relation to Gleason score, treatment and bone metastasis Biomed Pharmacother 2011 65 516 524 21993000 10.1016/j.biopha.2011.06.003

[bib48] Shiota M Yokomizo A Naito S Pro-survival and anti-apoptotic properties of androgen receptor signaling by oxidative stress promote treatment resistance in prostate cancer Endocr Relat Cancer 2012 19 R243 R253 23033314 10.1530/ERC-12-0232

[bib49] Selvendiran K Ahmed S Dayton A Ravi Y Kuppusamy ML Bratasz A HO-3867, a synthetic compound, inhibits the migration and invasion of ovarian carcinoma cells through downregulation of fatty acid synthase and focal adhesion kinase Mol Cancer Res 2010 8 1188 1197 20713491 10.1158/1541-7786.MCR-10-0201 PMC2941821

[bib50] Drayton RM The role of microRNA in the response to cisplatin treatment Biochem Soc Trans 2012 40 821 825 22817741 10.1042/BST20120055

[bib51] Blower PE Chung JH Verducci JS Lin S Park JK Dai Z MicroRNAs modulate the chemosensitivity of tumor cells Mol Cancer Ther 2008 7 1 9 18187804 10.1158/1535-7163.MCT-07-0573

[bib52] Weidhaas JB Babar I Nallur SM Trang P Roush S Boehm M MicroRNAs as potential agents to alter resistance to cytotoxic anticancer therapy Cancer Res 2007 67 11111 11116 18056433 10.1158/0008-5472.CAN-07-2858 PMC6070379

[bib53] Watahiki A Wang Y Morris J Dennis K O'Dwyer HM Gleave M MicroRNAs associated with metastatic prostate cancer PLoS One 2011 6 e24950 21980368 10.1371/journal.pone.0024950 PMC3184096

[bib54] Jenkins RB Qian J Lieber MM Bostwick DG Detection of c-myc oncogene amplification and chromosomal anomalies in metastatic prostatic carcinoma by fluorescence *in situ* hybridization Cancer Res 1997 57 524 531 9012485

[bib55] Iversen PL Arora V Acker AJ Mason DH Devi GR Efficacy of antisense morpholino oligomer targeted to c-myc in prostate cancer xenograft murine model and a Phase I safety study in humans Clin Cancer Res 2003 9 2510 2519 12855625

[bib56] Leonetti C Biroccio A D'Angelo C Semple SC Scarsella M Zupi G Therapeutic integration of c-myc and bcl-2 antisense molecules with docetaxel in a preclinical model of hormone-refractory prostate cancer Prostate 2007 67 1475 1485 17654511 10.1002/pros.20636

[bib57] Gao P Tchernyshyov I Chang TC Lee YS Kita K Ochi T c-Myc suppression of miR-23a/b enhances mitochondrial glutaminase expression and glutamine metabolism Nature 2009 458 762 765 19219026 10.1038/nature07823 PMC2729443

[bib58] Fontana L Fiori ME Albini S Cifaldi L Giovinazzi S Forloni M Antagomir-17-5p abolishes the growth of therapy-resistant neuroblastoma through p21 and BIM PLoS One 2008 3 e2236 18493594 10.1371/journal.pone.0002236 PMC2375057

[bib59] Sugimura K Miyata H Tanaka K Hamano R Takahashi T Kurokawa Y Let-7 expression is a significant determinant of response to chemotherapy through the regulation of IL-6/STAT3 pathway in esophageal squamous cell carcinoma Clin Cancer Res 2012 18 5144 5153 22847808 10.1158/1078-0432.CCR-12-0701

[bib60] Hebert C Norris K Scheper MA Nikitakis N Sauk JJ High mobility group A2 is a target for miRNA-98 in head and neck squamous cell carcinoma Mol Cancer 2007 6 5 17222355 10.1186/1476-4598-6-5 PMC1783857

