# Editorial Expression of Concern: USP2a alters chemotherapeutic response by modulating redox

**DOI:** 10.1038/s41419-025-08406-1

**Published:** 2026-01-13

**Authors:** B. Benassi, M. Marani, M. Loda, G. Blandino

**Affiliations:** 1 Unit of Radiation Biology and Human Health, ENEA-Casaccia, Rome, Italy; 2 Molecular Medicine Department, Italian National Cancer Institute Regina Elena, Rome, Italy; 3 Department of Medical Oncology and Center for Molecular Oncologic Pathology, Dana-Farber Cancer Institute, Harvard Medical School, Boston, MA USA; 4 Division of Cancer Studies, King’s College London, London, UK; 5 Translational Oncogenomics Unit, Italian National Cancer Institute Regina Elena, Rome, Italy

Editorial Expression of Concern to: *Cell Death and Disease* 10.1038/cddis.2013.289, published online 26 September 2013

The Editors-in-Chief are issuing this Editorial Expression of Concern to alert readers that concerns have been raised regarding some of the data reported in this article. In Supplementary Figure 1a, two β-Actin blots appear to have been duplicated from elsewhere in this article: β-Actin at 48h appears to be a duplicate of part of the B-actin blot in Figure 1c, and β-Actin at 72h appears to be a duplicate of part of the β-Actin blot in Figure 1b. The authors were contacted about these irregularities, but due to the age of this article were no longer able to provide full raw data for the impacted figures. Readers are therefore advised to interpret the data presented in this article with caution.

All authors agree with this Editorial Expression of Concern.

