# Supplementary material for: USP2a alters chemotherapeutic response by modulating redox
Source: Cell Death Dis. 2013 Sep 26;4(9):e812–. doi: 10.1038/cddis.2013.289 (PMC3789164; doi:10.1038/cddis.2013.289)
Supplement: Supplementary Figure 1 [file cddis2013289x1.ppt]

## Slide 1
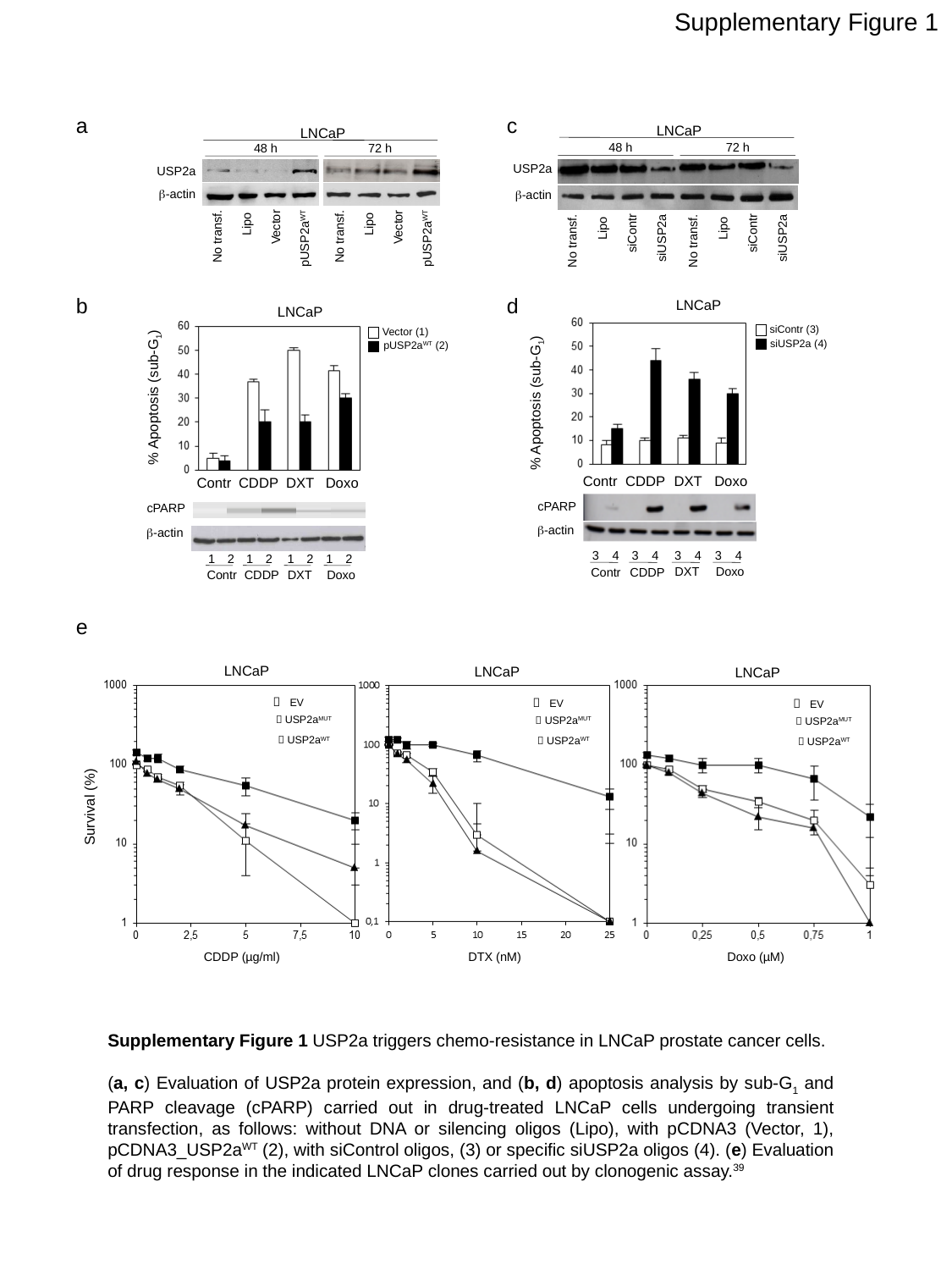

Supplementary Figure 1
a
c
LNCaP
72 h
48 h
USP2a
β-actin
Lipo
Lipo
siContr
siContr
siUSP2a
siUSP2a
No transf.
No transf.
LNCaP
72 h
48 h
USP2a
β-actin
Lipo
Lipo
Vector
Vector
No transf.
No transf.
pUSP2aWT
pUSP2aWT
b
d
LNCaP
LNCaP
siContr (3)
siUSP2a (4)
Vector (1)
pUSP2aWT (2)
% Apoptosis (sub-G1)
% Apoptosis (sub-G1)
DXT
Doxo
Contr
CDDP
DXT
Doxo
Contr
CDDP
cPARP
cPARP
β-actin
β-actin
3
4
3
4
3
4
3
4
DXT
Doxo
Contr
CDDP
1
2
1
2
1
2
1
2
DXT
Doxo
Contr
CDDP
e
LNCaP
 EV
 USP2aMUT
 USP2aWT
LNCaP
 EV
 USP2aMUT
 USP2aWT
LNCaP
 EV
 USP2aMUT
 USP2aWT
Survival (%)
CDDP (µg/ml)
DTX (nM)
Doxo (µM)
Supplementary Figure 1 USP2a triggers chemo-resistance in LNCaP prostate cancer cells.
(a, c) Evaluation of USP2a protein expression, and (b, d) apoptosis analysis by sub-G1 and PARP cleavage (cPARP) carried out in drug-treated LNCaP cells undergoing transient transfection, as follows: without DNA or silencing oligos (Lipo), with pCDNA3 (Vector, 1), pCDNA3_USP2aWT (2), with siControl oligos, (3) or specific siUSP2a oligos (4). (e) Evaluation of drug response in the indicated LNCaP clones carried out by clonogenic assay.39
