# Supplementary material for: USP2a alters chemotherapeutic response by modulating redox
Source: Cell Death Dis. 2013 Sep 26;4(9):e812–. doi: 10.1038/cddis.2013.289 (PMC3789164; doi:10.1038/cddis.2013.289)

## Slide 1
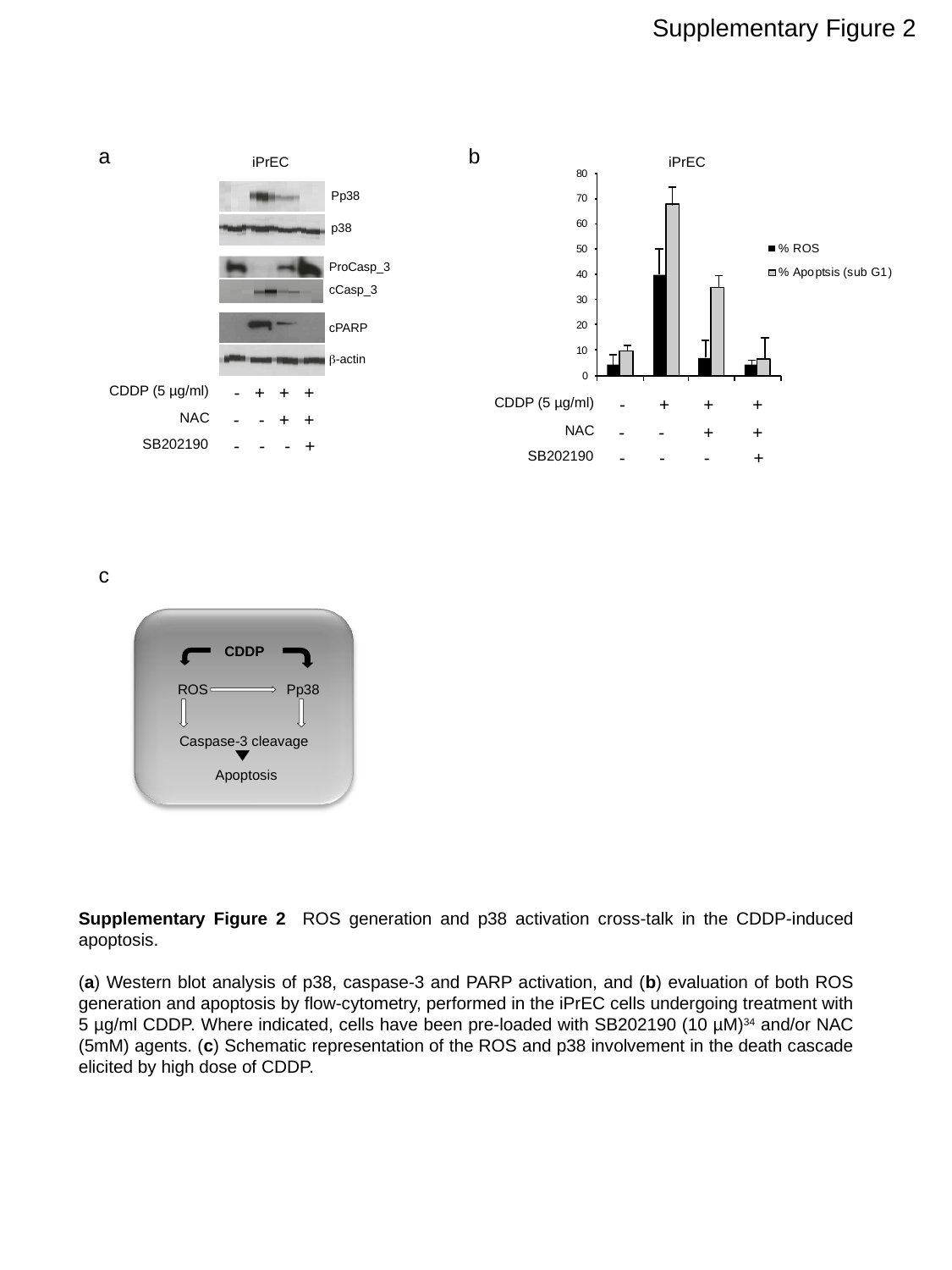

Supplementary Figure 2
a
b
iPrEC
iPrEC
Pp38
p38
ProCasp_3
cCasp_3
cPARP
β-actin
CDDP (5 µg/ml)
- + + +
NAC
- - + +
SB202190
- - - +
CDDP (5 µg/ml)
- + + +
NAC
- - + +
SB202190
- - - +
c
CDDP
ROS
Pp38
Caspase-3 cleavage
Apoptosis
Supplementary Figure 2 ROS generation and p38 activation cross-talk in the CDDP-induced apoptosis.
(a) Western blot analysis of p38, caspase-3 and PARP activation, and (b) evaluation of both ROS generation and apoptosis by flow-cytometry, performed in the iPrEC cells undergoing treatment with 5 µg/ml CDDP. Where indicated, cells have been pre-loaded with SB202190 (10 µM)34 and/or NAC (5mM) agents. (c) Schematic representation of the ROS and p38 involvement in the death cascade elicited by high dose of CDDP.

## Slide 2
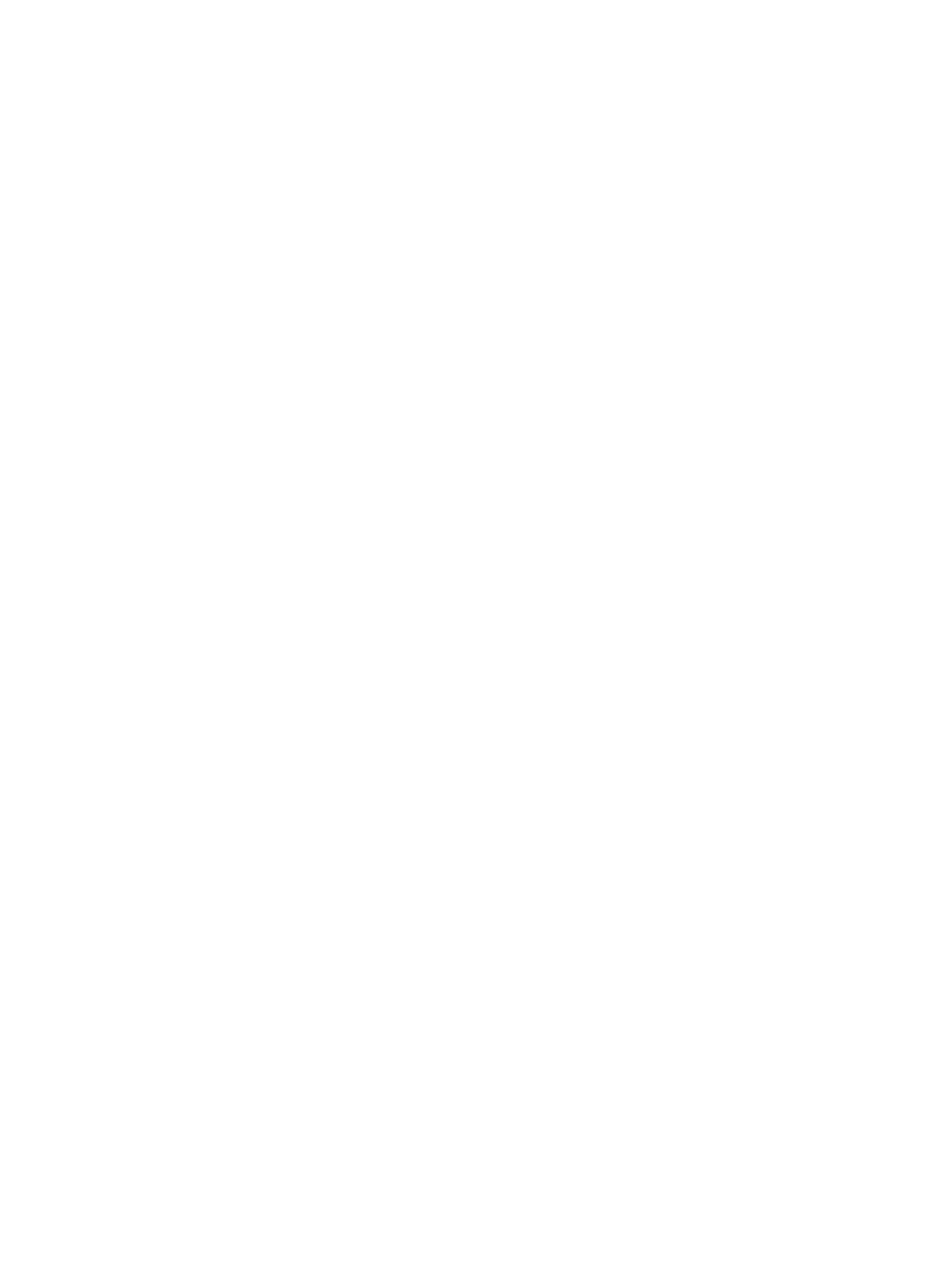

Supplement: Supplementary Figure 2 [file cddis2013289x2.ppt]
