# Supplementary material for: USP2a alters chemotherapeutic response by modulating redox
Source: Cell Death Dis. 2013 Sep 26;4(9):e812–. doi: 10.1038/cddis.2013.289 (PMC3789164; doi:10.1038/cddis.2013.289)
Supplement: Supplementary Figure 3 [file cddis2013289x3.ppt]

## Slide 1
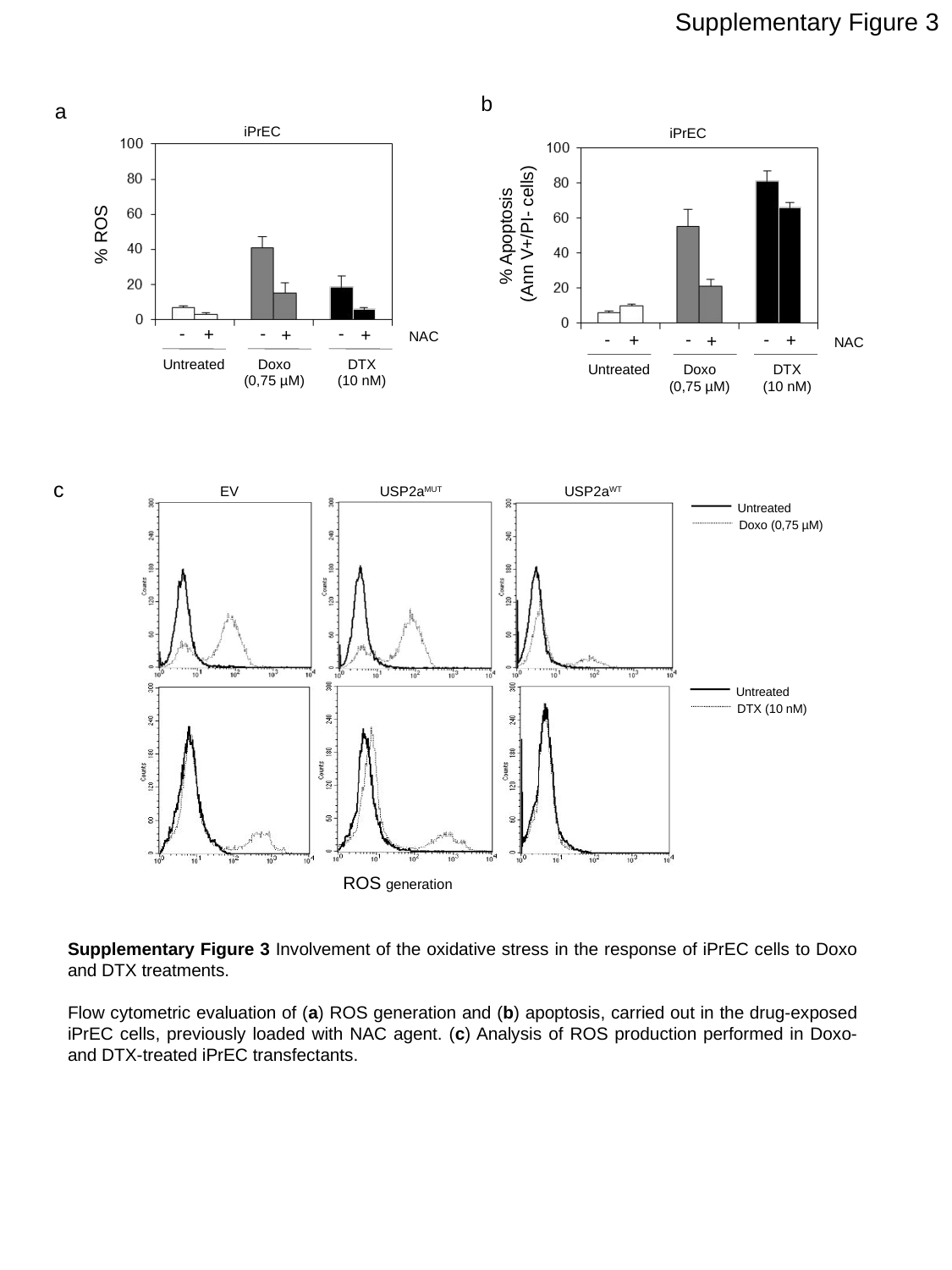

Supplementary Figure 3
b
iPrEC
% Apoptosis
 (Ann V+/PI- cells)
-
-
-
 +
 +
 +
NAC
Untreated
Doxo
(0,75 µM)
DTX
(10 nM)
a
iPrEC
% ROS
-
-
-
 +
 +
 +
NAC
Untreated
Doxo
(0,75 µM)
DTX
(10 nM)
c
EV
USP2aMUT
USP2aWT
ROS generation
Untreated
Doxo (0,75 µM)
Untreated
DTX (10 nM)
Supplementary Figure 3 Involvement of the oxidative stress in the response of iPrEC cells to Doxo and DTX treatments.
Flow cytometric evaluation of (a) ROS generation and (b) apoptosis, carried out in the drug-exposed iPrEC cells, previously loaded with NAC agent. (c) Analysis of ROS production performed in Doxo- and DTX-treated iPrEC transfectants.
