# Supplementary material for: USP2a alters chemotherapeutic response by modulating redox
Source: Cell Death Dis. 2013 Sep 26;4(9):e812–. doi: 10.1038/cddis.2013.289 (PMC3789164; doi:10.1038/cddis.2013.289)
Supplement: Supplementary Figure 4 [file cddis2013289x4.ppt]

## Slide 1
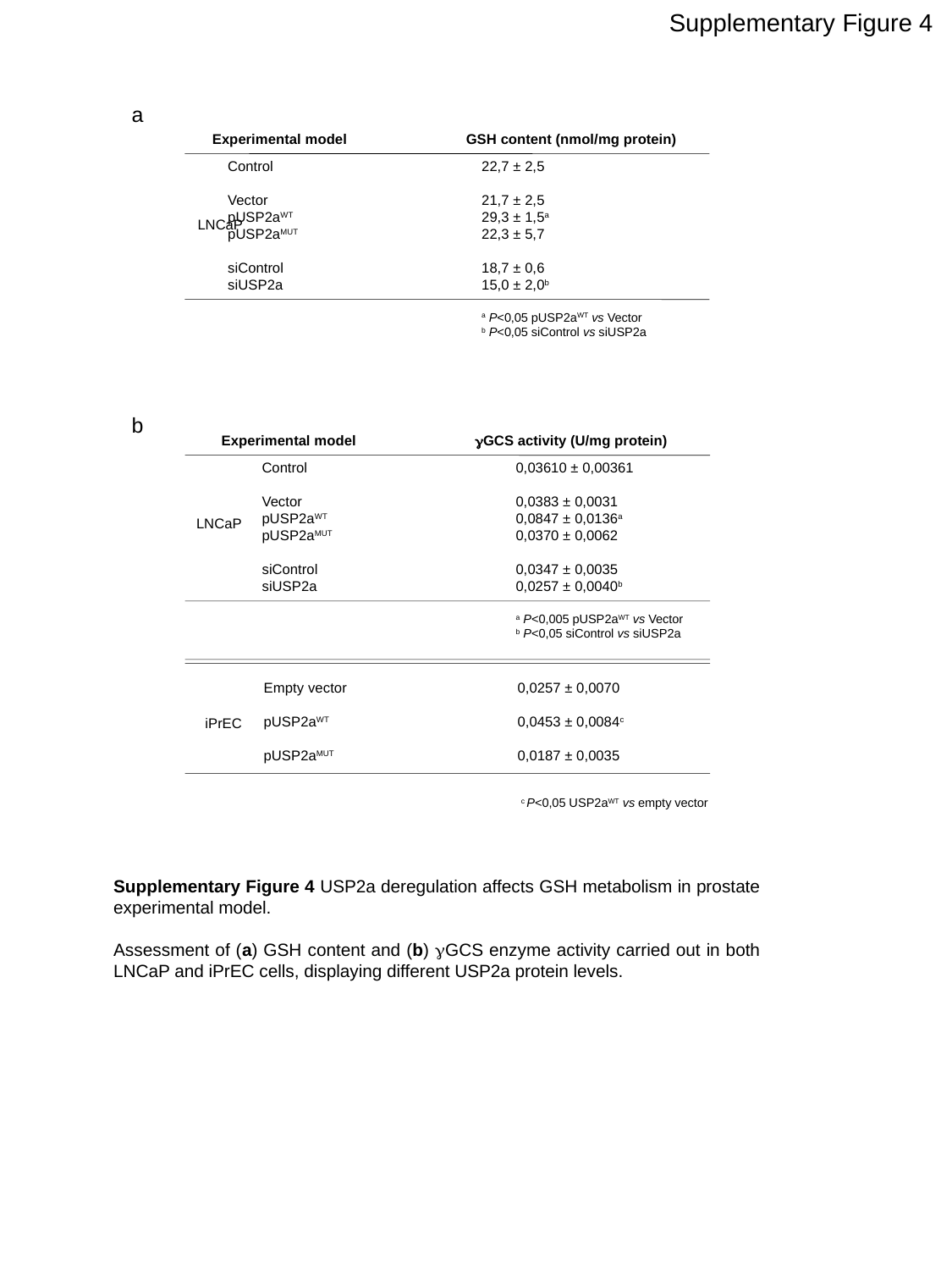

Supplementary Figure 4
a
Experimental model	GSH content (nmol/mg protein)
Control		22,7 ± 2,5
Vector 		21,7 ± 2,5
pUSP2aWT		29,3 ± 1,5a
pUSP2aMUT		22,3 ± 5,7
siControl		18,7 ± 0,6
siUSP2a		15,0 ± 2,0b
a P<0,05 pUSP2aWT vs Vector
b P<0,05 siControl vs siUSP2a
LNCaP
b
Experimental model 	γGCS activity (U/mg protein)
Control		0,03610 ± 0,00361
Vector 		0,0383 ± 0,0031
pUSP2aWT		0,0847 ± 0,0136a
pUSP2aMUT		0,0370 ± 0,0062
siControl		0,0347 ± 0,0035
siUSP2a		0,0257 ± 0,0040b
		a P<0,005 pUSP2aWT vs Vector
		b P<0,05 siControl vs siUSP2a
LNCaP
Empty vector		0,0257 ± 0,0070
pUSP2aWT		0,0453 ± 0,0084c
pUSP2aMUT		0,0187 ± 0,0035
		 c P<0,05 USP2aWT vs empty vector
iPrEC
Supplementary Figure 4 USP2a deregulation affects GSH metabolism in prostate experimental model.
Assessment of (a) GSH content and (b) γGCS enzyme activity carried out in both LNCaP and iPrEC cells, displaying different USP2a protein levels.
