# Supplementary material for: USP2a alters chemotherapeutic response by modulating redox
Source: Cell Death Dis. 2013 Sep 26;4(9):e812–. doi: 10.1038/cddis.2013.289 (PMC3789164; doi:10.1038/cddis.2013.289)
Supplement: Supplementary Figure 5 [file cddis2013289x5.ppt]

## Slide 1
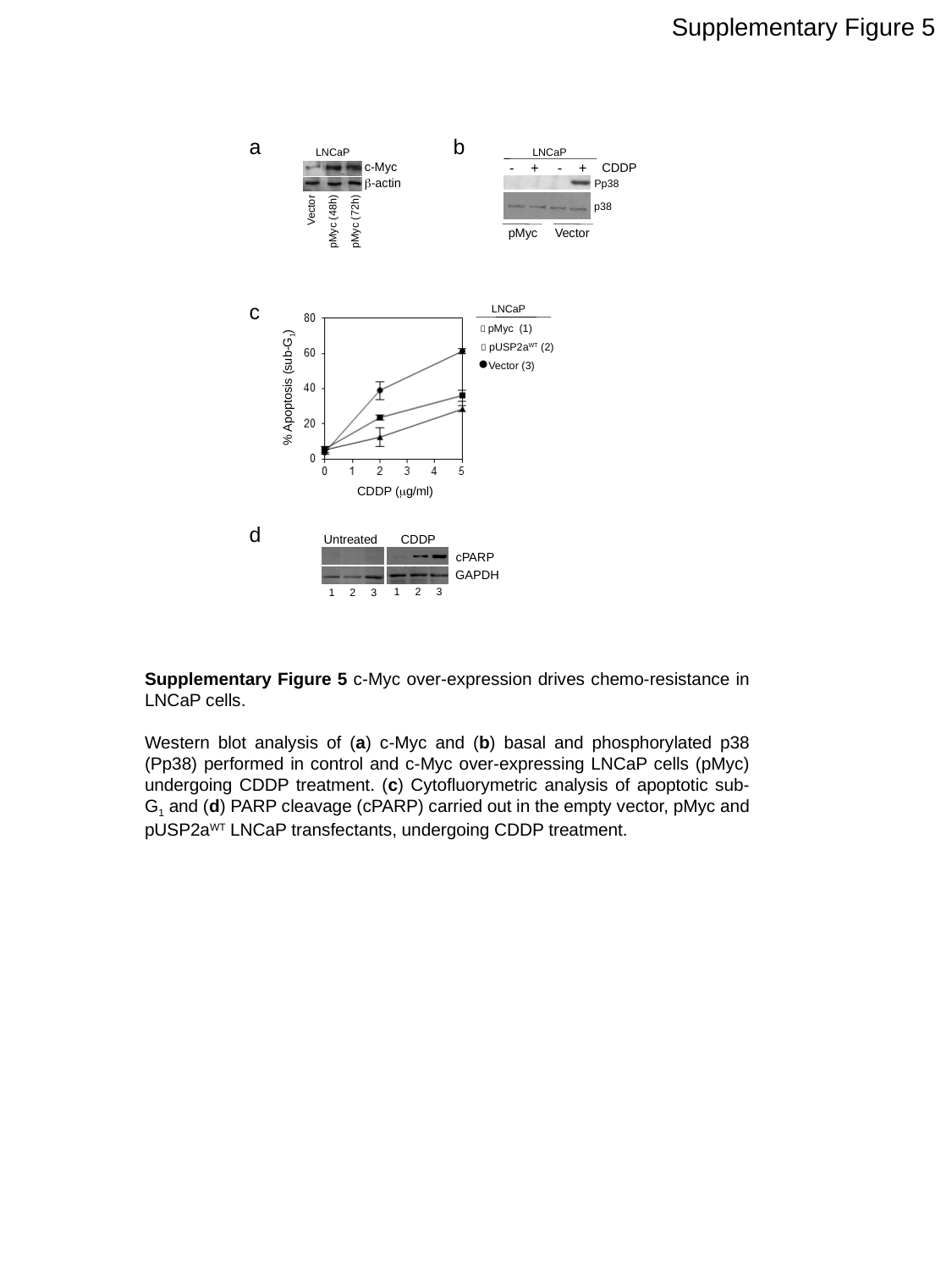

Supplementary Figure 5
a
b
LNCaP
LNCaP
-
+
-
+
c-Myc
CDDP
β-actin
Pp38
p38
Vector
pMyc (48h)
pMyc (72h)
pMyc
Vector
c
LNCaP
 pMyc (1)
 pUSP2aWT (2)
●Vector (3)
% Apoptosis (sub-G1)
CDDP (μg/ml)
d
Untreated
 CDDP
cPARP
GAPDH
1
2
3
1
2
3
Supplementary Figure 5 c-Myc over-expression drives chemo-resistance in LNCaP cells.
Western blot analysis of (a) c-Myc and (b) basal and phosphorylated p38 (Pp38) performed in control and c-Myc over-expressing LNCaP cells (pMyc) undergoing CDDP treatment. (c) Cytofluorymetric analysis of apoptotic sub-G1 and (d) PARP cleavage (cPARP) carried out in the empty vector, pMyc and pUSP2aWT LNCaP transfectants, undergoing CDDP treatment.
