# Supplementary material for: USP2a alters chemotherapeutic response by modulating redox
Source: Cell Death Dis. 2013 Sep 26;4(9):e812–. doi: 10.1038/cddis.2013.289 (PMC3789164; doi:10.1038/cddis.2013.289)
Supplement: Supplementary Figure 6 [file cddis2013289x6.ppt]

## Slide 1
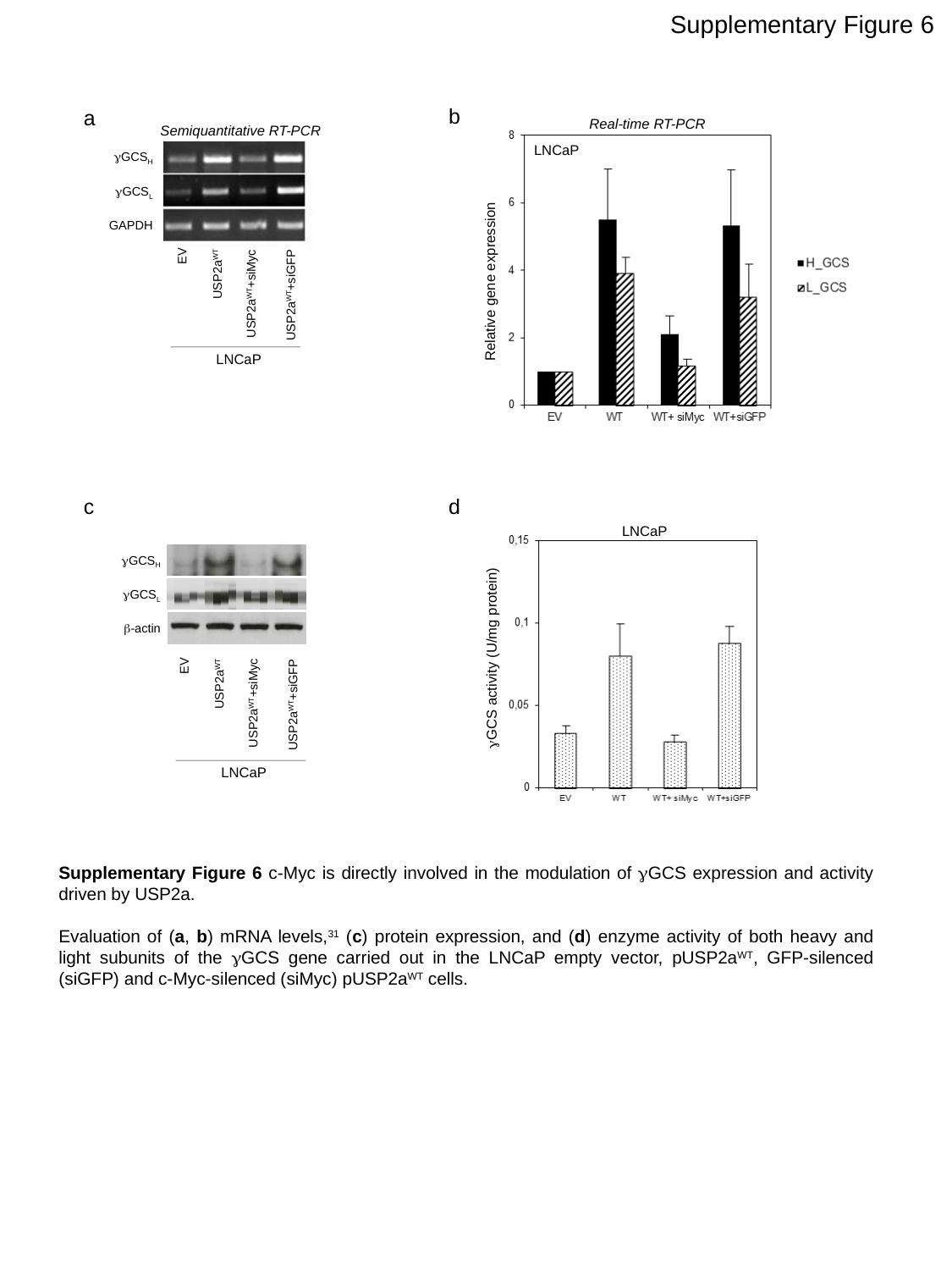

Supplementary Figure 6
b
a
Real-time RT-PCR
Semiquantitative RT-PCR
γGCSH
γGCSL
GAPDH
EV
USP2aWT
USP2aWT+siMyc
USP2aWT+siGFP
Relative gene expression
LNCaP
LNCaP
c
d
LNCaP
γGCS activity (U/mg protein)
γGCSH
γGCSL
β-actin
EV
USP2aWT
USP2aWT+siMyc
USP2aWT+siGFP
LNCaP
Supplementary Figure 6 c-Myc is directly involved in the modulation of γGCS expression and activity driven by USP2a.
Evaluation of (a, b) mRNA levels,31 (c) protein expression, and (d) enzyme activity of both heavy and light subunits of the γGCS gene carried out in the LNCaP empty vector, pUSP2aWT, GFP-silenced (siGFP) and c-Myc-silenced (siMyc) pUSP2aWT cells.
