# Supplementary material for: USP2a alters chemotherapeutic response by modulating redox
Source: Cell Death Dis. 2013 Sep 26;4(9):e812–. doi: 10.1038/cddis.2013.289 (PMC3789164; doi:10.1038/cddis.2013.289)
Supplement: Supplementary Figure 7 [file cddis2013289x7.ppt]

## Slide 1
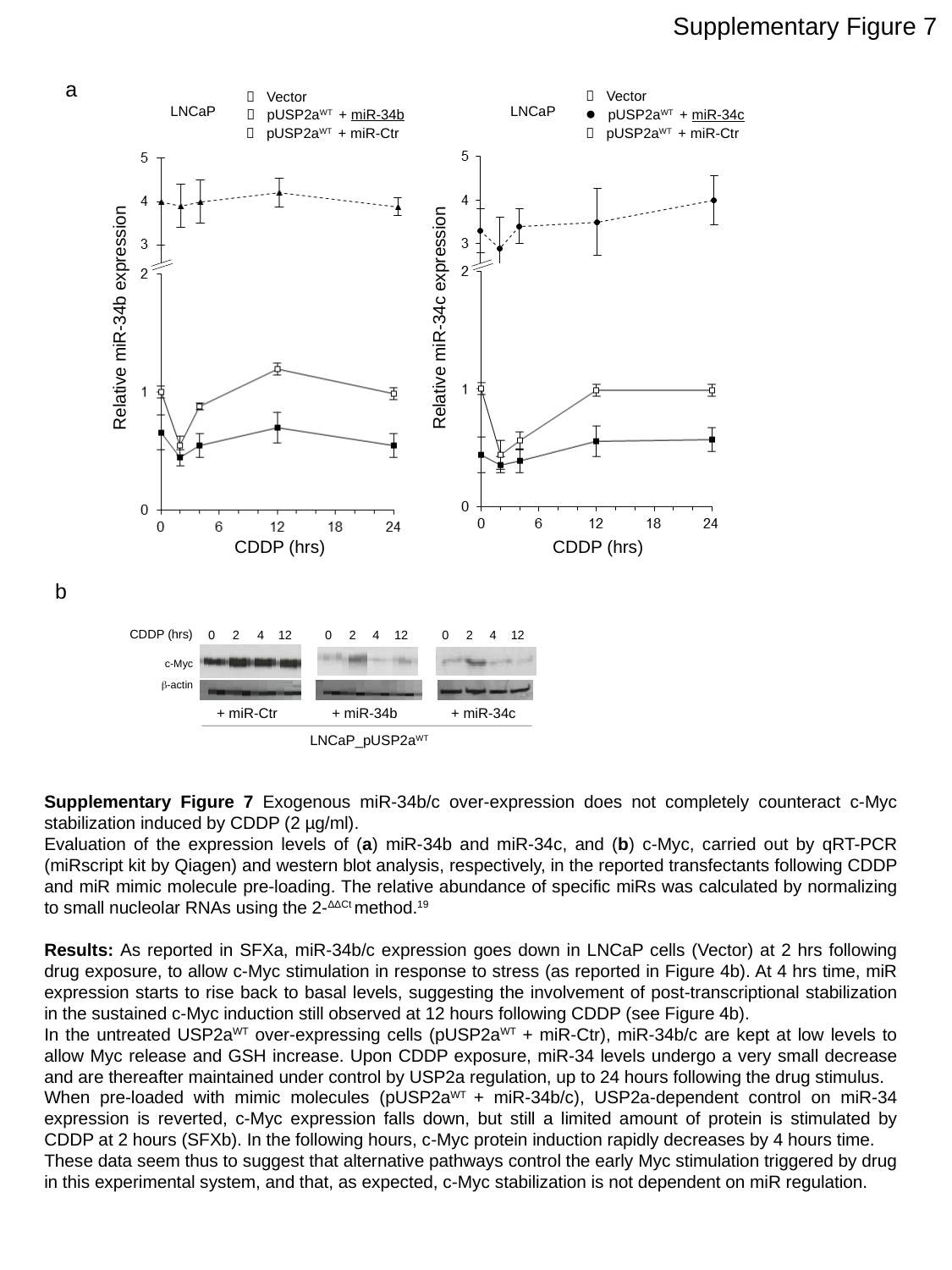

Supplementary Figure 7
a
 Vector
● pUSP2aWT + miR-34c
 pUSP2aWT + miR-Ctr
LNCaP
 Vector
 pUSP2aWT + miR-34b
 pUSP2aWT + miR-Ctr
LNCaP
Relative miR-34b expression
Relative miR-34c expression
CDDP (hrs)
CDDP (hrs)
b
CDDP (hrs)
0
2
4
12
0
2
4
12
0
2
4
12
c-Myc
β-actin
+ miR-Ctr
+ miR-34b
+ miR-34c
LNCaP_pUSP2aWT
Supplementary Figure 7 Exogenous miR-34b/c over-expression does not completely counteract c-Myc stabilization induced by CDDP (2 µg/ml).
Evaluation of the expression levels of (a) miR-34b and miR-34c, and (b) c-Myc, carried out by qRT-PCR (miRscript kit by Qiagen) and western blot analysis, respectively, in the reported transfectants following CDDP and miR mimic molecule pre-loading. The relative abundance of specific miRs was calculated by normalizing to small nucleolar RNAs using the 2-ΔΔCt method.19
Results: As reported in SFXa, miR-34b/c expression goes down in LNCaP cells (Vector) at 2 hrs following drug exposure, to allow c-Myc stimulation in response to stress (as reported in Figure 4b). At 4 hrs time, miR expression starts to rise back to basal levels, suggesting the involvement of post-transcriptional stabilization in the sustained c-Myc induction still observed at 12 hours following CDDP (see Figure 4b).
In the untreated USP2aWT over-expressing cells (pUSP2aWT + miR-Ctr), miR-34b/c are kept at low levels to allow Myc release and GSH increase. Upon CDDP exposure, miR-34 levels undergo a very small decrease and are thereafter maintained under control by USP2a regulation, up to 24 hours following the drug stimulus.
When pre-loaded with mimic molecules (pUSP2aWT + miR-34b/c), USP2a-dependent control on miR-34 expression is reverted, c-Myc expression falls down, but still a limited amount of protein is stimulated by CDDP at 2 hours (SFXb). In the following hours, c-Myc protein induction rapidly decreases by 4 hours time.
These data seem thus to suggest that alternative pathways control the early Myc stimulation triggered by drug in this experimental system, and that, as expected, c-Myc stabilization is not dependent on miR regulation.
